# The effectiveness and cost-effectiveness of treatments for idiopathic pulmonary fibrosis: systematic review, network meta-analysis and health economic evaluation

**DOI:** 10.1186/2050-6511-15-63

**Published:** 2014-11-19

**Authors:** Emma Loveman, Vicky R Copley, Jill L Colquitt, David A Scott, Andy J Clegg, Jeremy Jones, Katherine MA O’Reilly, Sally Singh, Claudia Bausewein, Athol Wells

**Affiliations:** Southampton Health Technology Assessments Centre (SHTAC), University of Southampton, Southampton, UK; Icon PLC, Oxford, UK; Mater Misericordiae University Hospital, Dublin, Ireland; University Hospitals of Leicester NHS Trust, Leicester, UK; Department of Palliative Medicine, University Hospital of Munich, Munich, Germany; Green Lane Hospital, London, UK

**Keywords:** Idiopathic pulmonary fibrosis, Systematic review, Meta-analysis, Cost-effectiveness

## Abstract

**Background:**

Idiopathic pulmonary fibrosis (IPF) is a life-limiting lung disease with considerable impact on patients and carers as the disease progresses. Currently few treatments are available. We aimed to evaluate the clinical and cost-effectiveness of available treatments for IPF.

**Methods:**

Systematic reviews of clinical effectiveness, quality of life and cost effectiveness were undertaken. Eleven bibliographic databases were searched from inception to July 2013 and studies were assessed for eligibility against a set of pre-defined criteria. Two reviewers screened references, extracted data from included studies and appraised their quality. An advisory group was consulted about the choice of interventions. A narrative review was undertaken and where feasible fixed effect and random effects meta-analysis were undertaken including a network meta-analysis (NMA).

A decision-analytic Markov model was developed to estimate cost-effectiveness of pharmacological treatments for IPF. Following best practice recommendations, the model perspective was of the national health service and personal social services, a discount rate of 3.5% for costs and health benefits was applied and outcomes were expressed as cost per quality adjusted life-year gained. Parameter values were obtained from the NMA and systematic reviews. Sensitivity analyses were undertaken.

**Results:**

Fourteen studies were included in the review of clinical effectiveness, of which one evaluated azathioprine, three N-acetylcysteine [NAC] (alone or in combination), four pirfenidone, one nintedanib, one sildenafil, one thalidomide, two pulmonary rehabilitation, and one a disease management programme. Study quality was generally good. Evidence suggests that some effective treatments are available. In NMA only nintedanib and pirfenidone show statistically significant improvements. The model results show increased survival for five pharmacological treatments (NAC triple therapy, inhaled NAC, nintedanib, pirfenidone, and sildenafil) compared with best supportive care, at increased cost. Only inhaled NAC was cost-effective at current willingness to pay thresholds but it may not be clinically effective.

**Conclusions:**

Few interventions have any statistically significant effect and the cost-effectiveness of treatments is uncertain. A lack of studies on palliative care approaches was identified and there is a need for further research into pulmonary rehabilitation and thalidomide in particular. A well conducted RCT on inhaled NAC therapy should also be considered.

**Electronic supplementary material:**

The online version of this article (doi:10.1186/2050-6511-15-63) contains supplementary material, which is available to authorized users.

## Background

Idiopathic pulmonary fibrosis (IPF) is a debilitating respiratory condition for which there is no cure. IPF is characterised by aberrant wound healing in which excessive (and perhaps abnormal) extracellular matrix is deposited in the lung thereby distorting the architecture and disrupting function. This lung injury and scarring eventually leads to a decline in lung function which culminates in respiratory failure [1]. Shortness of breath on exercise and a chronic dry cough are the prominent symptoms [2]. IPF is known to affect males more than females and in particular affects people over 60 years of age. The prevalence of IPF is increasing, although the cause of this increase is uncertain, [3, 4] and with a poor prognosis (estimated mean survival of between 2–5 years) IPF has become an area of focus with recent UK national guidelines on the diagnosis and management of IPF published, [5] and international consensus guidelines [6] due to be updated in 2014.

IPF is a difficult condition to manage, particularly in the latter stages. Early and accurate diagnosis is important to maximise the potential for a better outcome but there is an unmet need with few recommended treatments [5]. In the UK all patients should be given best supportive care (BSC) from the point of diagnosis, which includes information and support, symptom relief, management of co-morbidities, withdrawal of ineffective therapies and end-of-life care. In addition individuals should be assessed for pulmonary rehabilitation if appropriate and have a clinical nurse specialist available to them [5]. For those without contraindications lung transplantation should be considered as this is the only treatment shown to improve survival [7]. However, with donor organs being in short supply there is a need for alternative treatments that aim to modify the disease and prolong survival. Few treatments are available to the clinician and patient currently, and the evidence for the effectiveness of such treatments is unclear. We aimed to evaluate the current state of the clinical and cost-effectiveness of treatments for people with IPF through three systematic reviews, a network meta-analysis (NMA) and economic modelling.

## Methods

The *a priori* methods for this evidence synthesis are described in the research protocol which is registered with PROSPERO (reference: 42012002116). Search strategies were developed and applied to 11 electronic bibliographic databases (including the Cochrane library, MEDLINE and EMBASE) from inception to July 2013 with no language restrictions. Bibliographies of retrieved papers were screened and experts contacted to identify additional studies. Systematic reviews were undertaken of clinical effectiveness (including only randomised controlled trials [RCTs] and controlled clinical trials [CCTs]), economic evaluations and health related quality of life (HRQoL) studies. Eligible participants were those with a diagnosis of IPF and includable interventions were as deemed relevant by a clinical and patient advisory group. Best supportive care, placebo or any of the interventions were eligible as comparators and outcomes of relevance included measures of survival, measures of symptoms (breathlessness, cough), HRQoL, lung function, exercise performance, adverse events and measures of costs and cost-effectiveness. Studies reporting HRQoL in people with IPF were eligible for inclusion if they used either generic preference-based measures or the St Georges Respiratory Questionnaire (SGRQ) which is a disease specific instrument used in IPF. Other disease specific instruments were not eligible for inclusion as there are currently no methods to map results of these to utility measures required for economic evaluation.

Titles and abstracts were screened for potential eligibility by two reviewers using a pre-defined inclusion criteria, retrieved articles were assessed for eligibility, data were extracted and methodological quality assessed by one reviewer and checked by a second. Study quality was assessed using recognised methods [8–10]. For the review of clinical effectiveness we developed a check-list to assess the methodological quality of the studies based on the criteria recommended by the Centre for Reviews and Dissemination, [8] (Quality assessment/risk of bias section) and summarised the risk of bias (as per Cochrane collaboration recommendations [11]) within each study according to the risk of selection bias. We developed a check-list to assess the methodological quality of the cost effectiveness studies based on the check-list of Drummond and colleagues [9] and recommendations by Phillips and colleagues [10]. Data items extracted included study details (design, follow-up, funding), participant details (numbers, eligibility, characteristics), intervention details (including dose and duration of treatment), outcomes reported and results. Narrative syntheses were undertaken and in the review of clinical effectiveness meta-analysis was performed where appropriate with heterogeneity assessed. FVC was measured on two continuous scales and these were meta-analysed using the standardised mean difference (SMD). A NMA focusing on pharmacological treatments for IPF and assessing forced vital capacity (FVC) endpoints was undertaken [12].The NMA focused on FVC as it is correlated with disease progression [6] and was therefore relevant to the economic model. For FVC endpoints the NMA used the SMD in a Bayesian framework using code adapted from published sources [13]. Vague normal priors were used for the treatment effects and a vague uniform prior for the random effect standard deviation. Model code is provided in the Additional file 1. Fixed and random effects models were applied with best model fit determined using the deviance information criterion (DIC). The SMDs output from the NMA were then converted to log odds ratios using standard methods for ease of interpretation within the context of an NMA [14].

### Quality assessment/risk of bias

Was the method used to generate random allocations adequate?
Was the allocation adequately concealed?
Were the groups similar at the outset of the study in terms of prognostic factors
Was the care provider blinded?
Was the patient blinded?
Were outcome assessors blinded to the treatment allocation?
i) Were there any unexpected imbalances in drop-outs between groups? ii) If so, were they explained or adjusted for?
Is there any evidence to suggest that the authors measured more outcomes than they reported?
i) Did the analysis include an intention to treat analysis? ii) If so, was this defined?
i) Did the analysis account for missing data? ii) If so, were the methods appropriate?

A decision-analytic model was developed to compare the cost-effectiveness of pharmacological interventions in patients with initially unprogressed IPF. The model perspective is that of the UK National Health Service and Personal Social Services. The model structure was informed by the available literature and expert opinion on the clinical progression of the disease. It uses four distinct health states: unprogressed IPF; progressed IPF; lung-transplant; and dead. Health states except death are associated with a HRQoL utility and a cost estimate. Progression is defined by an absolute decline in FVC per cent predicted of ≥10% from a baseline (recently-diagnosed) value, based on the included RCTs. Published sources were used to inform the probability of a lung transplant; survival after lung transplant; and all-cause mortality by age. Acute exacerbations are not modelled as separate health states but are associated with a cost and utility decrement. Model cycle length is one month and a lifetime horizon of 30 years was adopted to capture all clinically and economically important events. A half-cycle correction is applied. Key assumptions are that all patients are in in the unprogressed state initially; those experiencing a ≥10% absolute decline in FVC% predicted are considered to be in the progressed health state; and treatment has a constant effect on relative rate of FVC% decline, FVC% predicted was used as a proxy for disease severity when assigning utilities to the health states. In addition, where treatment costs for any individual treatment were not available an assumed cost was used and tested in threshold analysis.

Treatment effects were obtained from the NMA. Utility values from the systematic review of HRQoL are applied to the modelled health states to estimate the benefits measured as quality adjusted life years (QALYs). Costs are included for treatments, treatment monitoring, acute exacerbations, lung transplant and adverse events, based on the UK health system. Future costs and benefits are discounted at 3.5% per annum. The outcome is reported as cost per QALY gained against the next best alternative treatment using incremental cost effectiveness ratios (ICERs). The model examines uncertainty in deterministic and probabilistic sensitivity analyses. Model validation was undertaken by checking structure, calculations and data inputs. In addition the advisory group reviewed the structure and internal consistency was examined by varying input values. Model results were compared with trial outputs and other publications [15–17].

## Results

### Clinical effectiveness

**Figure 1:**
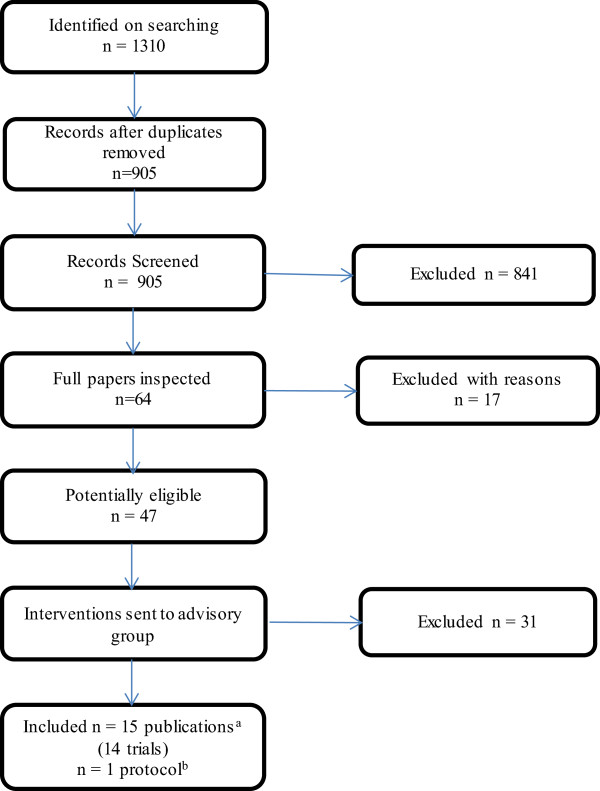
**Flow chart for the identification of studies in the clinical effectiveness review.** ^a^ Two RCTs were reported in one publication; two further trials each had two linked publications. ^b^ One trial was published as a protocol only.

Searches identified 905 unique references and 64 of these were retrieved after screening of titles and abstracts. Fourteen studies (13 RCTs and 1 CCT) were included (Figure 1).Four RCTs evaluated the use of pirfenidone, [15, 18, 19] three the use of n-acetylcysteine (alone or in combination), [20–22] one azathioprine, [23] one nintedanib, [16] one sildenafil, [24] one thalidomide, [25] one a pulmonary rehabilitation programme, [26] and one a disease management programme [27]. In addition one CCT of pulmonary rehabilitation was included [28]. This study was published in Polish and translation of key methods and results only were undertaken due to resource and time constraints. Therefore caution is recommended in interpreting our assessment of this study. No studies of palliative care interventions were identified that met the inclusion criteria. Study quality was generally good with a low risk of bias. Ten studies were undertaken in populations that would likely be classed as mild to moderate IPF [29]. The majority of these studies had reasonable sample sizes and duration of follow was between nine months and 16 months. Four studies were undertaken in populations that would be classed as moderate to severe IPF. Three of these were the non-pharmacological intervention studies, and one the drug sildenafil. Sample sizes were generally smaller in the non-pharmacological studies, and there was no long-term follow up. Across all studies the mean ages of participants ranged from approximately 54–69 years, the gender ratio of males to females was generally 3:1, and the duration of diagnosis tended to be between 1 and 3 years. In the ten studies in mild-to-moderate IPF the baseline FVC ranged between 65-90% and in the four studies in moderate-to-severe IPF this ranged from 55-70%. The populations were deemed to be reasonably similar to those seen in clinical practice by our advisory group. For further summary details see Table 1.

**Table 1:** **Study characteristics of included interventions**

Study and intervention details	Baseline characteristics	Outcomes	Risk of bias (selection bias)
**Pharmacological agents**			
**Azathioprine**			
Raghu et al. 1991 [21]	Mean Age: 56 years	*Primary outcomes:* not stated as primary or secondary: measurable change in lung function (FVC, DLCO, P[A-a]O2) at 12 months; survival	Unclear risk
*Country:* USA	M/F%: 55/45		
*Design:* RCT	Time since diagnosis: 2 years		
*Number of centres:* 2	FVC: 67%		
*Funding:* Grant from Virginia Mason ResearchCentre, Seattle, USA		*Length of follow-up*: 12 months	
*Interventions:*			
*1.* Prednisone and placebo, n = 13			
*2.* Prednisone and azathioprine, n = 14			
*Duration of treatment:* 12 months			
**BIBF-1120**			
Richeldi et al. 2011 [22]	Mean Age: 65 years	*Primary outcomes:* annual rate of decline in FVC	Low risk
*Country:* 25 countries including Italy, Mexico,	M/F%: 75/25		
Germany, USA, Korea, UK, France.	Time since diagnosis: 1.2	*Secondary outcomes: %* predicted FVC; DLCO; SpO2; TLC; 6MWT, SGRQ, decrease in FVC of more than 10% or more than 200 ml; SpO2 decrease of more than 4%; acute exacerbations; survival; death from a respiratory cause; adverse events	
*Design:* RCT (dose finding phase II study)	years		
*Number of centres:* 92	FVC: 80%		
*Funding:* supported by Boehringer Ingelheim			
*Interventions:*			
*1.* BIBF 1120 50 mg/day, n = 86			
*2.* BIBF 1120 50 mg twice per day (100 mg/day), n = 86		*Length of follow-up*: 54 weeks	
*3*. BIBF 1120 100 mg twice per day (200 mg/day), n = 86			
*4.* BIBF 1120 150 mg twice per day (300 mg/day), n = 85			
*5.* Placebo, n = 85			
*Duration of treatment:* 52 weeks			
**N-Acetylcysteine (alone or in combination)**			
Demedts et al. 2005 [18]	Mean Age: 63 years	*Primary outcomes:* absolute changes in VC and DLCO at 12 months	Low risk
*Country:* Belgium, France, Germany, Italy, Spain, the Netherlands	M/F%: 72/28		
*Design:* RCT	Time since diagnosis: 1.6 years	*Secondary outcomes: %* predicted VC, % predicted DLCO, alveolar volume change and % predicted, CRP score, dyspnoea score, maximum exercise indexes, HRCT outcomes, SGRQ, adverse events, withdrawals, and mortality	
*Number of centres:* 36	FVC: 66%		
*Funding:* sponsored by the Zambon group			
*Interventions:*			
*1.* N-acetylcysteine, prednisolone, azathioprine, n = 92 (80 analysed)			
*2.* Placebo, prednisolone, azathioprine, n = 90 (75 analysed)		*Length of follow-up*: 12 months	
*Duration of treatment:* not stated, assume 12 months.			
Raghu et al., (IPFCRN) 2012 [19]	Mean Age: 68 years	*Primary outcomes:* change in FVC at 60 weeks	Low risk
*Country:* USA	M/F%: 75/25		
*Design:* RCT (PANTHER study)	Time since diagnosis: 1 year	*Secondary outcomes:* rate of death, time until death, frequency of acute exacerbation, frequency of maintained FVC response, time to disease progression, clinical and physiological measures including: DLCO, 6MWT, CPI, UCSD SBQ, SGRQ, SF-36, EQ-5D. Adverse events.	
*Number of centres:* 25	FVC: 71%		
*Funding:* grants from the NHLBI; the Cowlin Family fund. NAC and placebo donated by Zambon			
*Interventions:*			
*1.* N-acetylcysteine and placebo (data not presented in article as ‘ongoing’ data collection), n = 81			
*2.* N-acetylcysteine/prednisolone/azathioprine, n = 77		*Length of follow-up*: 60 weeks in the planned analysis. The study was stopped early. The mean follow-up was 32 weeks.	
*3.* Placebo, n = 78			
*Duration of treatment:* up to 60 weeks			
Homma et al. 2012 [20]	Mean Age: 68 years	*Primary outcomes:* absolute change in FVC at 48 weeks	Unclear risk
*Country:* Japan	M/F%: 76/24		
*Design:* RCT	Time since diagnosis: 3 years		
*Number of centres:* 27	FVC: 89%	*Secondary outcomes:* changes in lowest aterial O2 saturation, 6MWT distance, PFT parameters (VC, % predicted VC, TLC, % predicted TLC, DLCO, predicted DLCO), serum markers of pneumocyte injury; disease progression as determined by HRCT; subjective changes in symptoms such as dyspnoea, adverse events.	
*Funding:* grant from Ministry of Health, Labour and Welfare			
*Interventions:*			
*1.* N-acetylcysteine inhaled, n = 51 (38 analysed)			
*2.* Control, n = 49 (38 analysed) *Duration of treatment:* 48 weeks			
		*Length of follow-up*: 48 weeks	
**Pirfenidone**			
Noble et al., 2011 [15]	Mean Age: 67 years	*Primary outcomes:* change in per cent predicted FVC	Low risk
Capacity study 006	M/F%: 72/28		
*Country:* Australia, Belgium, Canada, France, Germany, Ireland, Italy, Mexico, Poland, Spain, Switzerland, UK, USA	Time since diagnosis: ≤1		
	year: 59%	*Secondary outcomes:* categorical FVC (5-point scale), progression-free survival, worsening IPF, dyspnoea, 6MWT distance, worst peripheral oxygen saturation (SpO2) during the 6MWT, per cent predicted DLco, fibrosis, mortality.	
	FVC: 74%		
*Design:* RCT			
*Number of centres:* 110 centres			
*Funding:* InterMune			
*Interventions:*			
*1.* Pirfenidone 2403 mg/day, n = 171		*Length of follow-up:* 72 weeks from the date the last patient was enrolled.	
*2.* Placebo, n = 173			
*Duration of treatment:* 72 weeks			
Noble et al., 2011 [15]	Mean Age: 66 years	*Primary outcomes:* change in per cent predicted FVC	Low risk
Capacity study 004	M/F%: 71/29		
*Country:* Australia, Belgium, Canada, France, Germany, Ireland, Italy, Mexico, Poland, Spain, Switzerland, UK, USA	Time since diagnosis: ≤1		
	year: 48%	*Secondary outcomes:* categorical FVC (5-point scale), progression-free survival, worsening IPF, dyspnoea, 6MWT distance, worst peripheral oxygen saturation (SpO2) during the 6MWT, per cent predicted DLco, mortality.	
	FVC: 75%		
*Design:* RCT			
*Number of centres:* 110 centres			
*Funding:* InterMune			
*Interventions:*			
*1.* Pirfenidone 2403 mg/day, n = 174		*Length of follow-up:* 72 weeks from the date thelast patient was enrolled	
*2.* Pirfenidone 1197 mg/day, n = 87			
3. Placebo, n = 174			
*Duration of treatment:* 72 weeks			
Taniguchi et al., 2010 [16]	Mean Age: 65 years	*Primary outcomes:* change in vital capacity to week 52	Unclear risk
*Country:* Japan	M/F%: 78/22		
*Design:* RCT	Time since diagnosis: <1		
*Number of centres:* 73	year: 37%	*Secondary outcomes:* Progression-free survival time, change in lowest SpO2 during the 6MET. Pa,O2, PA-a,O2, TLC and DLCO, acute exacerbation, markers of interstitial pneumonias, symptoms.	
*Funding:* public sector grants. Drug and placebo from Shionogi & Co, Ltd.	FVC: 78%		
*1.* Pirfenidone 1800 mg/day, n = 108			
*2.* Pirfenidone 1200 mg/day, n = 55			
*3.* Placebo, n = 104		*Length of follow-up:* 52 weeks	
*Duration of treatment:* 52 weeks			
Azuma et al., 2005 [17]	Mean Age: 64 years	*Primary outcomes:* change in the lowest SpO2 during the 6MET	Unclear risk
*Country:* Japan	M/F%: 90/10		
*Design:* RCT	Time since diagnosis: <1		
*Number of centres:* 25	year: 22%	*Secondary outcomes:* resting PFTs while breathing air (VC, TLC, DLCO PaO2), disease progression by HRCT patterns, acute exacerbation, serum markers of pneumocyte damage, QoL	
*Funding:* Shionogi & co, Ltd	FVC: 80%		
*Interventions:*			
*1.*Pirfenidone 1800 mg/day, n = 73			
*2.* Placebo, n = 36			
*Duration of treatment:* 9 months			
		*Length of follow-up*: minimum of 9 months	
**Thalidomide**			
Horton *et al.,* 2012 [24]	Mean Age: 68 years	*Primary outcomes:* cough-specific quality of life (CQLQ)	Low risk
*Country:* USA	M/F%: 78/22		
*Design:* randomised cross-over trial	Time since diagnosis: 1.7		
*Number of centres:* one	years	*Secondary outcomes:* cough, respiratory quality of life.	
*Funding:* Celgene Corporation	FVC: 70%		
*Interventions:*			
*1. Thalidomide,* n = 23		*Method of assessing outcome:* Cough-specific quality of life measured by CQLQ. Cough measured by 10 cm VAS. Respiratory quality of life measured by SGRQ.	
*2. Placebo,* n = 23			
*Duration of treatment:* 12 weeks each treatment with a 2 week washout period between treatments.			
		*Length of follow-up:* 12 weeks.	
**Sildenafil (severe IPF)**			
Zisman and colleagues IPFCRN, 2010 [23]	Mean Age: 69 years	*Primary outcomes:* presence or absence of an improvement of at least 20% in the 6MWT distance at 12 weeks.	Unclear risk
*Country:* USA	M/F%: 84/16		
*Design:* RCT	Time since diagnosis: 1.9		
*Number of centres:* 14	years		
*Funding:* NHLBI; the Cowlin Fund (Chicago Community trust); Pfizer; Masimo.	FVC: 57%	*Secondary outcomes:* changes in the 6MWT distance, degree of dyspnoea, quality of life, FVC, DLCO, arterial partial pressure of oxygen and arterial oxygen saturation, and the alveolar-arterial oxygen gradient while breathing ambient air, adverse events, hospitalisations, death.	
*1.* Sildenafil, n = 89			
*2.* Placebo, n = 91			
*Duration of treatment:* 12 weeks.			
		*Length of follow-up*: 12 weeks	
**Non-pharmacological interventions**			
**Disease management programme/Pulmonary Rehabilitation**			
Lindell et al. 2010 [26]	Mean Age: 66 years	*Primary outcomes:* Not specified as primary or secondary outcomes. Dyspnoea (UCSDSBQ); Anxiety (BAI); Depression (BDI-II); Stress (PSS); QoL (SF-36)	Unclear risk
*Country:* USA	M/F%: 76/24		
*Design:* RCT	Time since diagnosis: NR		
*Number of centres:* one	FVC: >55: 70%		
*Funding:* Fairbanks-Horix Foundation			
*Interventions:*		Length of follow-up: Unclear	
*1.* Program to Reduce IPF Symptoms and Improve Management (PRISIM), n = 10 pairs			
*2.* Usual care, n = 11 pairs			
*Duration of treatment:* 6 weeks			
Jastrzebski et al. 2008 [27]	Mean Age: 56 years	*Primary outcomes:* not specified as primary or secondary. Dyspnoea (oxygen cost diagram, baseline dyspnoea index). QoL (SF-36), 6MWT (distance, dyspnoea in Borg’s scale), maximal inspiratory pressure, lung function tests (IC, TLC, VC, FEV1, DLCOSB, DLCO/VA).	High risk
*Country:* Poland	M/F%: 64/36		
*Design:* CCT	Time since diagnosis: >2		
*Number of centres:* one	years		
*Funding:* not translated	FVC: 68%		
*Interventions:*			
*1.* Inspiratory muscle training, n = 16			
*2.* Control, n = 14		Length of follow-up: 12 weeks	
*Duration of treatment:* 12 weeks (two six week cycles)			
Nishiyama et al. 2008 [25]	Mean Age: 66 years	*Primary outcomes:* not specified as primary or secondary. Pulmonary function tests (FVC, FEV1, TLC, PaO2,PaCO2,) DLCO, 6MWT; BDI; SGRQ	Unclear risk
*Country:* Japan	M/F%: 76/24		
*Design:* RCT	Time since diagnosis: NR		
*Number of centres:* one	FVC: 67%		
*Funding:* Japanese ministry of health, labor and welfare		Length of follow-up: 10 weeks after the start of the programme.	
*Interventions:*			
*1.* Pulmonary rehabilitation programme (PRP), n = 15 (13 analysed)			
*2.* Control, n = 15			
*Duration of treatment:* 10 week programme.			

Results for the clinical effectiveness of the five pharmacological interventions in patients with mild to moderate IPF were mixed. In clinical practice azathioprine is only used in restricted circumstances, in one RCT [23] azathioprine and prednisolone led to an improvement in survival compared with placebo and prednisolone when an age adjusted analysis was used. There was no effect on lung function. This trial had an unclear risk of bias, a small sample size, and it is uncertain whether all patients had a diagnosis of IPF based on current recommendations. Consequently, caution is recommended when interpreting results. Nintedanib 300 mg/day was more favourable than placebo on some FVC measures, acute exacerbations and mortality, however, the primary outcome of annual rate of decline in FVC was not statistically significant [16]. Treatment with NAC was evaluated in three studies, [20–22] in combination with azathioprine and prednisolone in two (triple therapy) and in an inhaled format in one. Study results were mixed and establishing the stand-alone effect of NAC is difficult due to the differences between the three studies. There was no benefit from triple therapy on FVC compared to placebo in one trial, however, there was a benefit on vital capacity (VC) when compared to azathioprine and prednisolone in another. The treatment effect of inhaled NAC was not statistically different from that of a control group (p = 0.05). The study using inhaled NAC had an unclear risk of bias which should be considered when interpreting results. Four RCTs [15, 18, 19] evaluated the use of pirfenidone and meta-analysis shows that pirfenidone appears to demonstrate a significant effect on FVC when compared to placebo (SMD 0.24, 95% CI 0.06, 0.41, p = 0.008). This should be cautiously interpreted as the outcomes pooled were different, the timing of assessment varied (from 48 weeks to 72 weeks) and there was moderate statistical heterogeneity (I^2^ = 45%). The effect of pirfenidone on secondary outcomes was more uncertain. Thalidomide was assessed in those with cough in a small crossover RCT [25]. HRQoL outcomes related to cough were improved with thalidomide compared to placebo. There is no evidence relating to any subgroups in any of the published studies.

One study [24] assessed sildenafil for those with moderate to severe IPF. No statistically significant benefit of sildenafil was seen on the primary outcome, a 20% improvement on the six minute walk test. Results on secondary outcomes were mixed with some favourable to sildenafil but others being not statistically significant.

Adverse events were generally mild to moderate and reasonably well balanced between the treatments and controls with the exception of thalidomide which led to a greater proportion of people experiencing at least one adverse event (77%) than the placebo treated participants (22%) [25]. Severe adverse events appeared to be more common in one study in those treated with triple therapy [21].

**Figure 2:**
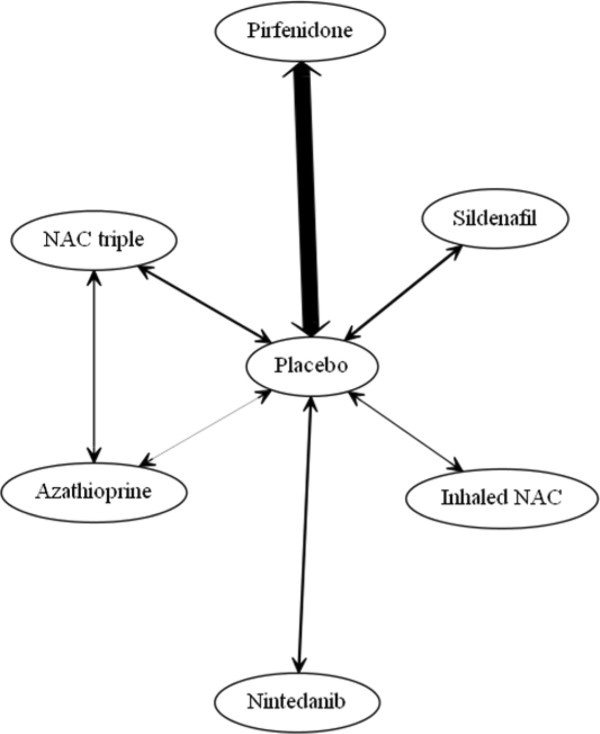
**Evidence network for FVC endpoint.** Legend: The width of the lines connecting treatment pairs is proportional to the number of participants within each trial comparison.

Ten studies of pharmacological interventions were included in NMA; the resulting evidence network is shown in Figure 2. Thalidomide was excluded as the focus of treatment is not on lung function. Inhaled NAC was considered separately from triple therapy owing to its different method of administration. Both direct and indirect evidence was used to assess the treatment effect compared to placebo. Only the fixed effect results for nintedanib and pirfenidone were statistically significant, odds ratios for reducing the rate of decline in FVC compared to placebo are shown in (Table 2). The random effects model did not demonstrate a better fit than the fixed effect model and there was no evidence of inconsistency within the evidence network. A head-to-head comparison of nintedanib versus pirfenidone suggested a trend favouring nintedanib, but this was not statistically significant and should be cautiously interpreted in the light of the various differences between the studies (Table 2). Further trial evidence could be used to test this further.

**Table 2:** **NMA Fixed effects results, SMDs converted to log odds ratios for slowing the decline in FVC**

			Log odds ratios			Odds ratios		
Comparator (vs. placebo)	N studies	Total N participants	Mean	95% CrI		Mean	95% CrI	
Azathioprine	1	19	0.44	-0.30	1.19	1.56	0.74	3.29
Nintedanib	1	170	-0.97	-1.52	-0.41	0.38	0.22	0.66
NAC triple therapy	2	294	-0.06	-0.60	0.48	0.94	0.55	1.62
Inhaled NAC	1	76	-0.42	-1.24	0.40	0.66	0.29	1.49
Pirfenidone	4	1006	-0.39	-0.62	-0.16	0.68	0.54	0.85
Sildenafil	1	180	-0.12	-0.65	0.41	0.89	0.52	1.51
*Head-to-head comparison*								
Nintedanib vs. Pirfenidone	-0.58			-1.18	0.03	0.56	0.31	1.03

Three non-pharmacological intervention studies compared a pulmonary rehabilitation programme or disease management programme to control interventions in moderate-to-severe IPF. Results from the two pulmonary rehabilitation studies were inconclusive; some outcomes were favourable to pulmonary rehabilitation but not others. The risk of bias was uncertain and endpoints were only assessed immediately after the interventions finished. Limited evidence of the effectiveness of a disease management programme was demonstrated. This study had an uncertain risk of bias and follow-up was immediate.

### Cost effectiveness and HRQoL systematic reviews

One economic evaluation was identified, which examined a testing strategy prior to treatment with triple therapy but did not examine the cost-effectiveness of treatment. The systematic review of HRQoL included 23 studies that examined HRQoL using either a generic preference-based tool (EuroQol five dimensions [EQ-5D], Short Form-36 [SF-36]) or a disease specific instrument (SGRQ) that could be mapped to utility for the economic model. Results varied between the studies, given the different populations under study and the different measures and time points of measurement, however, results generally showed that IPF has an adverse effect on HRQoL which increases with severity.

### Cost-effectiveness of pharmacological treatments for IPF

The baseline risk of disease progression in the unprogressed state was taken from two of the pirfenidone trials included in the systematic review of clinical effectiveness [15] as the population most closely met the model definition of unprogressed IPF and the RCTs had large sample sizes. The probabilities of progression free survival were obtained from the Kaplan Meier survival curve published for the pooled placebo population of two RCTs [15]. Parametric survival curves were fitted to this curve in Stata using maximum likelihood estimation in order to extrapolate beyond the 72 weeks follow-up. Exponential, Weibull, loglogistic, lognormal and Gompertz parametric models were examined. Goodness of fit was assessed using the Akaike Information Criterion (AIC). The Weibull model was selected because of the balance of good fit (AIC) and face validity (comparison of predicted survival with known survival in IPF). In the progressed state the monthly probability of death was taken from the overall survival curve in a recent study reporting survival for those experiencing a 10% or greater decline in FVC [30]. This study was an observational study but followed a larger sample than other published studies that differentiate survival by FVC decline and was deemed the most appropriate for the evaluation. Five parametric survival curves were fitted using the distributions noted above. The exponential distribution was selected (using AIC and face validity) to extrapolate beyond the five years of observed data.Probabilities of acute exacerbations in both unprogressed and progressed health states, and probabilities of lung transplant and survival from lung transplant, were taken from published sources.

HRQoL utility values were applied to each of the alive health states. These were not differentiated by treatment; the impact of treatment on utility was assumed to occur because of delay to disease progression which the model accounts for. The HRQoL values used for the unprogressed and progressed IPF health states were taken from EQ-5D values reported in two trials included in the systematic reviews of clinical effectiveness and HRQoL [21, 24]. These trials were conducted by the same clinical network and it is likely that the estimates of EQ-5D are consistent and can therefore be contrasted in our economic model. The utility values applied in the model can be seen in Table 3. Utility associated with lung transplant is taken from a UK-based study [31] which assessed HRQoL using the EQ-5D. We weighted the utility to account for a greater proportion of single lung transplants in the IPF population than the proportion seen in the study, based on clinical opinion. Utility data for acute exacerbations were not identified in the literature and therefore a utility decrement was applied that was in line with decrements seen in those with asthma and chronic obstructive pulmonary disease (Table 3). Sensitivity analyses were used to test these utility decrements.

**Table 3:** **EQ-5D utility values by model health state**

Model health state	EQ-5D (SD)
Unprogressed IPF (corresponds with an FVC ~72%)	0.80 (0.20)
Progressed IPF (corresponds with an FVC ~59%)	0.74 (0.19)
Lung transplant	
0-6 months after transplant	0.71 (0.38)
7-18 months after transplant	0.72 (0.31)
19-36 months after transplant	0.70 (0.33)
>36 months after transplant	0.68 (0.38)
Acute exacerbation decrement	0.20 (not available)

Five types of cost are considered in the economic model. The costs associated with each treatment were made up of the costs of the drug and the monitoring costs associated with the treatment. Dose information and unit costs were taken from published sources where available [32, 33]. Unit costs and data sources were unavailable for nintedanib and an assumed cost was used which was also subject to full sensitivity analysis. Hospital admission costs arising from acute exacerbations were estimated from NHS reference costs for all treatments as no treatment-specific acute exacerbation costs were available (£1361.04) [34]. Ongoing non-pharmacological treatment costs for management of the condition were included and covered annual home oxygen costs (£824.30) and the costs of long-term oxygen monitoring (£173.94) for the progressed IPF state. The costs associated with lung transplant were calculated using NHS reference costs [34] excluding outpatient procedures (£35,468.61). Costs of adverse events were attributed to the pharmacological interventions. Based on the incidence of serious adverse events seen in the trials included in the systematic review of clinical effectiveness, costs were applied for each event per patient in the first cycle of the model [34].

The model base-case results show increased survival for five of the treatments compared with BSC, at increased cost (see Table 4). The combination of azathioprine and prednisolone is dominated by BSC (treatment is more costly and less effective than BSC). Triple therapy is associated with an ICER of £41,811 per QALY gained when compared to BSC. Inhaled NAC is associated with an ICER of £5,037 per QALY gained when compared to BSC. Sildenafil, pirfenidone and nintedanib are not cost-effective at a willingness to pay (WTP) threshold of £30,000/QALY when compared to BSC. Therefore only one treatment, inhaled NAC, is cost-effective at a £30,000 WTP threshold, but its treatment effect is not statistically significant in the RCT, a small study with undetermined risk of bias.

**Table 4:** **Summary of base case results**

Treatment	Total costs (£)	Total QALYs	ICER vs. BSC (£/QALY)	ICER vs. next best option (£/QALY)
BSC	3,084	2.98	-	-
Azathioprine & prednisolone	4,313	2.66	Dominated	Dominated
NAC triple therapy	5,021	3.03	41,811	Extended Dominance
Inhaled NAC	5,029	3.37	5,037	5,037
Sildenafil	12,008	3.11	68,116	Dominated
Pirfenidone	70,118	3.34	190,146	Dominated
Nintedanib	139,613	4.01	132,658	209,246

NB: Nintedanib uses an assumed cost.

Deterministic and probabilistic sensitivity analyses tested uncertainty in model parameter values, including costs and probabilities of acute exacerbation and lung transplant. Treatment effects and utilities were also varied in sensitivity analyses. The parameters were varied between the 2.5^th^ and 97.5^th^ percentiles of the mean value and analyses found that results were generally robust but were particularly sensitive to changes in the value of the treatment effect parameters.

The monthly cost of nintedanib was assumed (£3,274). Results demonstrate that, given a WTP of £30,000 per QALY, nintedanib must cost less than £736 per month to be considered as the cost-effective treatment option compared to BSC and pirfenidone.

## Discussion

We systematically reviewed evidence for the clinical effectiveness of six pharmacological interventions and two non-pharmacological interventions for IPF. Participants in most of these studies would likely be classed as having mild-to-moderate IPF and generally were similar to those seen in clinical practice. There was a range of treatments under investigation in these trials with only one treatment, pirfenidone, providing evidence from more than one trial that was suitable for a formal meta-analysis to be undertaken. The outcomes reported in these studies differed however, and therefore caution is required when considering the results of the meta-analysis and the narrative synthesis of each of the included studies. In a network meta-analysis of the pharmacological treatments only pirfenidone and nintedanib had a statistically significant treatment effect, reducing the rate of decline in FVC compared to placebo. One pharmacological treatment was excluded from the network meta-analysis as the focus of treatment was not on lung function but on the symptom cough. In this study thalidomide appeared to improve cough, and quality of life compared with a placebo treatment. Results of three studies investigating two non-pharmacological treatments show mixed results and it is therefore unclear how effective these interventions are. There are differences between the studies in terms of the interventions, the participants, and the outcomes reported together with study design issues (e.g. short follow-up) and uncertain risk of bias that may account for some of the results seen.

Evidence from systematic reviews of cost effectiveness and HRQoL identified one economic evaluation of limited relevance and 23 HRQoL studies. These latter studies varied in their populations and study methods but generally showed that IPF has an adverse effect on HRQoL compared to population norms, and that HRQoL diminishes as IPF progresses. A new decision analytic health economic model was developed to assess the cost-effectiveness of six pharmacological treatments for IPF. Results show increased survival for five of the treatments compared with best supportive care, at increased cost. Only one treatment, inhaled NAC, is cost-effective at a WTP threshold of £30,000 but no statistically significant treatment effect was seen in the RCT or our NMA. Probabilistic sensitivity analysis showed that inhaled NAC has a 65% probability of being cost-effective if a decision threshold of £20,000 per QALY gained is used. Although pirfenidone and nintedanib achieve a statistically significant treatment effect in NMA they each have a probability of 0% of being cost-effective at a threshold of £30,000 per QALY. This is based on an assumed cost for nintedanib. A sensitivity analysis indicated that nintedanib must cost less than £736 per month to be considered cost-effective.

The past few years have seen an increasing interest in the management of IPF, with pharmacological companies evaluating a range of potential interventions, and a number of influential bodies producing guidelines. However, this systematic review demonstrates that at present there are few treatments which have any effect on surrogate outcomes which can be linked through evidence to patient related outcomes such as mortality. The findings of our research also suggest that under current willingness to pay thresholds only one treatment is likely to be cost effective, although, general recommendations cannot be made due to limitations in the evidence base. In terms of a cure it is considered that lung transplantation is the only intervention available which has curative intent. However, no evidence on lung transplant was eligible for inclusion in this evidence synthesis and so this could not be evaluated formally. There is also a scarcity of studies on interventions for symptom management and palliative care in IPF despite this being a recommended approach in recent clinical guidance [5].

No previous systematic reviews have included all potentially relevant treatments for IPF, and there has only been limited economic evaluation previously. Our results are useful to clinicians and patients, and complement recent national guidance by NICE in the UK [5]. In addition to standard synthesis we undertook a network meta-analysis to compare the pharmacological therapies to a common comparator. Our results show that only two treatments (nintedanib and pirfenidone) significantly slow the decline in FVC compared to placebo under a fixed effect model. However, with few studies it was not possible to fully explore heterogeneity within these data and the results should be cautiously interpreted. We also undertook an illustrative analysis comparing nintedanib with pirfenidone through an indirect comparison. While this showed a trend favouring nintedanib, it was not statistically significant and should be interpreted with caution until such time that a more complete analysis can be undertaken on more robust data. We identified a number of ongoing trials of potential relevance. Our evidence synthesis has highlighted the current evidence base in which these new trials can be contextualised once they report.

There were numerous differences between the studies included in this review. However we applied a rigorous approach to the inclusion, quality assessment and data synthesis of the studies (laid out in a research protocol), to ensure that our work was as unbiased as possible. Our research was guided by an advisory group from its initiation, in particular to ensure that the interventions included were appropriate to current or future management in the NHS. We ensured that only the highest quality studies were included to limit uncertainty in the results.

Our study has several limitations. The meta-analysis and NMA used the standardised mean difference to express findings from studies on a common scale. In this case we combined mean change in FVC% predicted with absolute change in FVC, albeit the former is adjusted for certain baseline characteristics, and this should be considered when interpreting the results. Many of the included studies compared treatments to placebo. Few direct comparisons were identified and results of an indirect comparison via the NMA approach are presented. However there are known limitations to the use of indirect comparisons which should also be considered in interpreting our findings [35]. The economic model assumes that treatments have a constant effect on the relative rate of FVC% decline compared to BSC, but treatment may in fact become less effective with time or as the condition progresses. (This would make the treatments less cost-effective than shown in our results.) Finally, because of limitations in the data the absolute decline in FVC% predicted was used as the measure of disease progression in the model. It is possible that use of this measure may introduce bias because the starting FVCs of patients (which might vary widely) is not taken into account.

## Conclusions

This evidence synthesis reports on the effectiveness of a range of interventions for IPF and complements recent UK guidance [5]. The current evidence suggests that there are currently few treatments which are clinically and cost-effective. Pirfenidone and nintedanib offer the potential for hope to sufferers and their clinicians, however, their cost-effectiveness is likely to be prohibitive. This research has thoroughly examined the current evidence and can be seen as a platform from which the clinical importance of newer treatments can be assessed when ongoing trials report. The systematic review has highlighted the need for further research into interventions to help alleviate or control symptoms of this debilitating condition, in particular pulmonary rehabilitation programmes and thalidomide. Given the results of our study and the weaknesses of the inhaled NAC trial, a well-designed RCT of inhaled NAC should also be considered; our search of ongoing RCTs failed to identify any such studies currently underway.

## Electronic supplementary material

Additional file 1: **NMA model code.** Random effects model code for the NMA. (DOCX 14 KB)

## Abbreviations

BSC: Best supportive care
CCT: Controlled clinical trial
EQ-5D: EuroQol five dimensions
FVC: Forced vital capacity
HRQoL: Health related quality of life
ICER: Incremental cost-effectiveness ratio
IPF: Idiopathic pulmonary fibrosis
UK: United Kingdom
NAC: N-acetylcysteine
NHS: National health service
NICE: National institute for health and care excellence
NMA: Network meta-analysis
QALY: Quality adjusted life Year
RCT: Randomised controlled trial
SD: Standard deviation
SF-36: Short Form-36
SMD: Standardised mean difference
VC: Vital capacity
WTP: Willingness to pay.
