# Supplementary material for: The effectiveness and cost-effectiveness of treatments for idiopathic pulmonary fibrosis: systematic review, network meta-analysis and health economic evaluation
Source: BMC Pharmacol Toxicol. 2014 Nov 19;15:63. doi: 10.1186/2050-6511-15-63 (PMC4247619; doi:10.1186/2050-6511-15-63)
Supplement: Supplementary file 1 — Additional file 1: NMA model code. Random effects model code for the NMA. (DOCX 14 KB) [file 40360_2014_347_MOESM1_ESM.docx]

Additional file 2.pdf

Title of data: NMA model code

Description of data: Random effects model code for the NMA

# Random effects model for two-arm trials (Dias et al, 2012)

model{ # *** PROGRAM STARTS

for(ii in 1:ns2) { # LOOP THROUGH 2-ARM STUDIES

y[ii,2] ~ dnorm(delta[ii,2],prec[ii,2]) # normal likelihood for 2-arm trials

var[ii,2] <- pow(se[ii,2],2) # calculate variances

prec[ii,2] <- 1/var[ii,2] # set precisions

dev[ii,2] <- (y[ii,2]-delta[ii,2])*(y[ii,2]-delta[ii,2])*prec[ii,2] #Deviance contribution

delta[ii,2] ~ dnorm(md[ii,2],tau) # trial-specific treat effects distributions

md[ii,2] <- d[t[ii,1]] - d[t[ii,2]] # mean of treat effects distributions

}

totresdev <- sum(dev[,2]) #Total Residual Deviance

d[1]<-0 # treatment effect is zero for reference treatment

for (kk in 2:nt){ d[kk] ~ dnorm(0,.0001) } # vague priors for treatment effects

sd ~ dunif(0,5) # vague prior for between-trial SD

tau <- pow(sd,-2) # between-trial precision = (1/between-trial variance)

} # *** PROGRAM ENDS
